# SART3, regulated by p53, is a biomarker for diagnosis, prognosis and immune infiltration in hepatocellular carcinoma

**DOI:** 10.18632/aging.204978

**Published:** 2023-08-24

**Authors:** Jusen Nong, Kejian Yang, Tianman Li, Chenlu Lan, Xin Zhou, Junqi Liu, Haixiang Xie, Jianzhu Luo, Xiwen Liao, Guangzhi Zhu, Tao Peng

**Affiliations:** 1 Department of Hepatobiliary Surgery, The First Affiliated Hospital of Guangxi Medical University, Nanning 530021, People’s Republic of China; 2 Key Laboratory of Early Prevention and Treatment for Regional High Frequency Tumor, Guangxi Medical University, Ministry of Education, Nanning 530021, People’s Republic of China; 3 Department of Hepatobiliary Surgery, The Sixth Affiliated Hospital of Guangxi Medical University, Yulin 537000, People’s Republic of China

**Keywords:** squamous cell carcinoma antigen recognized by T cells 3 (SART3), hepatocellular carcinoma (HCC), prognosis, *TP53* mutation, immune checkpoint, immune infiltration

## Abstract

Objective: This study aimed to investigate the role of squamous cell carcinoma antigen recognized by T cells 3 (SART3) in hepatocellular carcinoma (HCC).

Methods: SART3 expression and prognostic value were analyzed in TCGA and GEO datasets. The diagnostic value and prognostic significance of SART3 were determined using immunohistochemistry in the Guangxi cohort. The whole-exome mutation spectrum of SART3 was analyzed in high and low expression groups in both TCGA and Guangxi cohorts. The biological functions of the SART3 gene were validated through *in vitro* experiments using small interfering RNA technology to downregulate SART3 expression in HCC cell lines.

Results: SART3 expression was significantly higher in HCC tissues than in adjacent noncancerous liver tissues in TCGA, GEO and Guangxi cohorts. High expression of SART3 was significantly associated with poor prognosis in HCC patients. In TCGA and Guangxi cohorts, the expression of SART3 in the TP53 mutation group was significantly higher than that in the non-mutation group. Downregulation of SART3 expression significantly inhibited the migration and proliferation of HCC cells. SART3 may be involved mainly in immune infiltration of Th2 cells and macrophages in HCC. Additionally, SART3 can upregulate the expression of immune checkpoints (PD-L1 and TIM-3) and predict potential therapeutic agents for HCC.

Conclusion: The findings of this study demonstrate the diagnostic and prognostic value of SART3 in HCC. SART3 may be associated with immune infiltration of Th2 cells and macrophages in HCC, highlighting its potential role in the development and progression of HCC.

## INTRODUCTION

Primary liver cancer (PLC) is a frequently occurring malignancy with a continuously increasing incidence in recent years. According to the Global Cancer Observatory (GLOBOCAN) report [1], there were 900,000 new cases and 830,000 deaths due to liver cancer worldwide in 2020, emphasizing the high mortality rate of the disease. Hepatocellular carcinoma (HCC) is the most prevalent type of primary liver cancer, accounting for approximately 75%–85% of all pathological subtypes of PLC [2]. Despite the availability of treatment options such as surgical intervention, immunotherapy, targeted therapy, and hepatic artery infusion chemotherapy (HAIC), HCC patients still face a high five-year recurrence rate of approximately 70% [3]. Late-stage diagnosis in the majority of HCC patients precludes the use of radical resection. Thus, the development of innovative therapeutic approaches for advanced HCC patients are necessary. The immune microenvironment is known to play a pivotal role in tumor progression, invasion, recurrence, and metastasis [4, 5]. Immune-related genes play a crucial role in the tumor immune microenvironment and their comprehensive understanding may reveal novel insights into tumor progression, leading to the identification of potential prognostic biomarkers and Therapeutic targets.

Squamous cell carcinoma antigen recognized by T cells 3 (SART3) is a crucial regulator of pre-mRNA splicing. It is also known as Shearing factor 3 or HIV-1 Tat-interacting protein (TIP110). During the shedder recovery phase, SART3 binds and recombines U4/U6 small ribonucleoprotein (snRNP) and then binds to U6 snRNP to carry out its regulatory function [6, 7]. Besides its role in pre-mRNA splicing, SART3 is a multifunctional nuclear protein involved in regulating various cellular processes, including the cell cycle [8], mRNA synthesis [9, 10], tumor immune infiltration [11, 12], stem cell proliferation and differentiation, and maintenance of pluripotency [13, 14]. Multiple studies suggest that SART3 is closely associated with cancer. De Troyer et al. [15] reported that SART3 promotes tumor cell activity, while Timani et al. [16] found that SART3/Tip110 upregulates interleukin-8 to promote melanoma development and invasion. High expression of SART3 has been identified in colorectal cancer, and Sasatomi et al. [17] proposed that it could be a target gene for guiding immunotherapy in this cancer type.

The present findings suggest a close association between SART3 and tumor development as well as immune infiltration. Nonetheless, the precise mechanism underlying the functional role of SART3 in HCC remains to be elucidated.

## MATERIALS AND METHODS

### Data from public data platforms

Transcriptomic data pertaining to 33 human cancers were procured from the Cancer Genome Atlas (TCGA) and GTEx, which are publicly available data platforms hosted on the UCSC XENA platform (https://xenabrowser.net/datapages/) [18]. Furthermore, clinical data, survival data, and somatic mutation data of LIHC were acquired from TCGA, and the maftools R package was utilized to visualize somatic mutation data [19]. The GSE14520, GSE76427, GSE121248, and GSE62232 datasets were retrieved from the publicly accessible Gene Expression Omnibus (GEO) platform (https://www.ncbi.nlm.nih.gov/geo/) [20]. Lastly, we obtained data on SART3 amplification, truncation mutation, and splice mutation from the cBioPortal for Cancer Genomics platform for three HCC datasets (MERiC, Nat Commun/TCGA, Firehose Legacy/AMC, Hepatology 2014) [21]. We also acquired data on HCC drug sensitivity from the GDSC (Genomics of Drug Sensitivity in Cancer) database.

### Immunohistochemistry (IHC)

Paired tumor and paraneoplastic tissues from 70 HCC patients as well as tumor tissues from 16 HCC patients who were surgically resected were collected from the First Affiliated Hospital of Guangxi Medical University from September 2016 to September 2018 and 2020, respectively. The former samples were used to evaluate the diagnostic value and prognostic significance of SART3 in HCC, while the latter were subjected to next-generation sequencing (NGS) at GENETRON (Beijing, China) or REXPLO (Nanning, China) to examine the correlation between SART3 and TP53 mutations. The immunohistochemical techniques employed in this study were based on previous research [22]. Two independent pathologists evaluated and scored the immunohistochemical staining. Staining intensity was graded from 0 to 3, with 0 indicating no staining, 1 indicating weak staining, 2 indicating moderate staining, and 3 indicating strong staining. The percentage of positively stained cells was also taken into account, with scores of 1, 2, 3, and 4 assigned to tissues with 1%–25%, 26%–50%, 51%–75%, and 76%–100% positive cells, respectively [23]. The final staining score for each tissue was calculated as the product of the area score and the intensity score. In this study, a polyclonal antibody targeting SART3 (Proteintech, Wuhan, China) was used at a concentration of 1:400. Ethical approval was obtained from the First Affiliated Hospital of Guangxi Medical University prior to conducting the study to ensure adherence to ethical principles and guidelines (No. 2022-KY-E-159).

### Identification of differentially expressed genes

A DESeq2 R package [24] was employed to screen for differentially expressed genes (DEGs) in the TCGA database between samples of HCC with high and low expression levels of SART3, using a cut-off value of 50%. From the identified DEGs, the top 50 were selected for further analysis and presented through a heat map to enhance visual interpretation.

### Functional enrichment analysis and gene set enrichment analysis

We employed the ClusterProfiler (version 3.14.3) package in R to perform gene set enrichment analysis (GSEA) and functional enrichment analysis, aiming to identify the pathways and biological functions that are associated with the DEGs and SART3-related genes. The annotation of the genes was carried out using the Gene Ontology (GO) terms and the Kyoto Encyclopedia of Genes and Genomes (KEGG) pathways [25].

### Immune infiltration analysis

The evaluation of immune infiltration requires the use of TIMER, which is a publicly available data platform that investigates the relationship between gene expression levels and multiple immune cells [26, 27]. In this study, we employed TIMER, ssGSEA, and Cibersort to assess the correlation between SART3 expression levels and the extent of immune infiltration across different immune cell types.

### HCC cell lines

The Hep3B and Huh-7 cell lines were supplied by The National Collection of Authenticated Cell Cultures, while the HepG2 and SNU-449 cell lines were obtained from ATCC: The Global Bioresource Center. Prof. Lu Guo-dong, from the School of Public Health at Guangxi Medical University, provided the PLC/PRF/5 and MHCC-97H cell lines.

### Downward adjustment of SART3

The small interfering RNA (siRNA) designed to interfere with SART3 was synthesized by GenePharma (Suzhou, China) and the specific sequences utilized are detailed in Supplementary Table 1. The siRNA was then transfected into cells utilizing the Lipofectamine 3000 system (Invitrogen, USA), in accordance with the manufacturer’s instructions.

### Reverse transcription-polymerase chain reaction (RT-PCR)

RT-PCR was executed through implementation of a PrimeScript™ RT reagent Kit with gDNA Eraser (Takara, Beijing, China) for the purpose of genomic DNA erasure and reverse transcription, followed by utilization of a TB Green^®^ Premix Ex Taq™ in accordance with the manufacturer’s instructions.

### Proliferation assay

A proliferation assay was performed using Huh-7 and SUN-449 cells. These cells were inoculated into five identical 96-well plates after 48 hours of transfection with siRNA, with six replicate wells set up for each group. Following cell apposition, one plate was removed and designated as 0 h. CCK-8 reagent was added to each well, and the plates were then incubated in a suitable environment for two hours. Subsequently, the absorbance was measured at 450 nm. This procedure was repeated every 24 hours, and the results were collected for five days. Finally, the growth curve of the cells was plotted based on the collected data.

### Wound-healing and trans-well assays

Huh-7 and SUN-449 cells were seeded into 6-well plates with 3 replicate wells for each group. Upon reaching 90% confluence, a 200 μl pipette tip was used with a straightedge to slip-scrape the cell layer for Wound-Healing assay. Photomicrographs were captured and recorded at 0 h, followed by subsequent 24 h intervals. For Trans-well assay, 1 × 10^4^ cells were seeded into Trans-well chambers and adding 700 μl medium containing serum in a 24-well plate. After 48 h, the upper layer of cells was removed with a cotton swab, and the remaining lower layer was fixed in paraformaldehyde and stained with crystal violet. Photomicrographs were captured and recorded for further analysis.

### Statistical analysis

R 4.2.3 was utilized to perform statistical analyses and visualization. The expression of SART3 was compared between tumor and paraneoplastic tissues through Student’s *t*-test. Both the Kaplan-Meier curve and Cox regression model were employed for survival analysis (cut-off value of 50%). The association between the two groups was evaluated by Spearman’s correlation test. ROC curve for assessing the diagnostic capability of SART3. An area under the curve (AUC) value greater than 0.8 was deemed indicative of acceptable discrimination [28]. Statistical significance was determined by a *p*-value less than 0.05.

### Availability of data and materials

The data sets used and/or analyzed in this study are available from the corresponding authors upon reasonable request.

## RESULTS

### Elevated expression of SART3 in HCC

**Figure 1:**
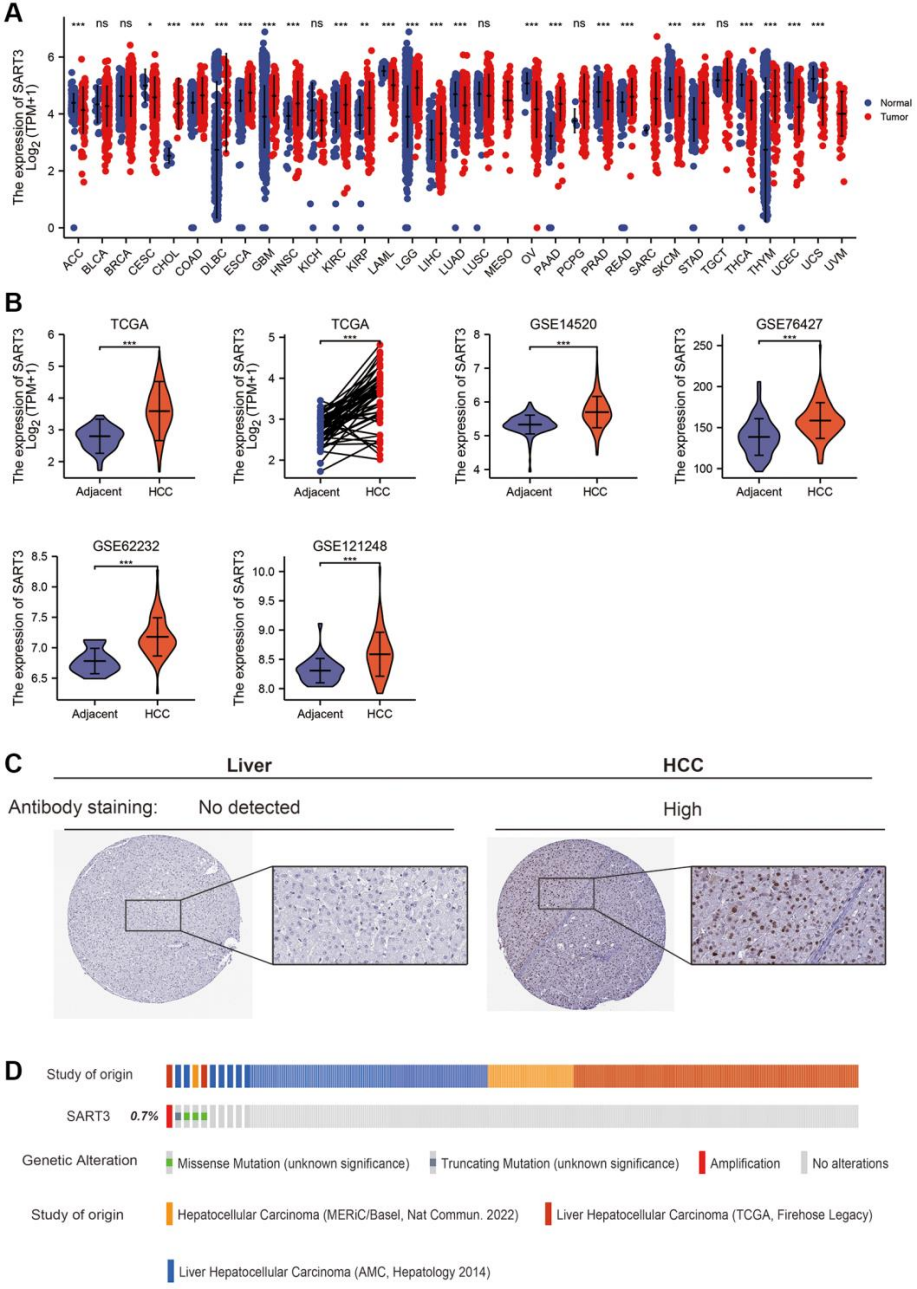
**SART3 is highly expressed in the HCC transcriptome and proteome.** (**A**) SART3 expression levels in 31 different human cancers. Panels (**B**) present the expression levels of SART3 in various datasets, including TCGA LIHC dataset, GSE14520 dataset, GSE76427 dataset, GSE62232 dataset, and GSE121248 dataset. (**C**) Validation of SART3 expression levels in the HCC proteome in the Human Protein Atlas database. (**D**) Distribution of genomic changes of SART3 in cBioPortal OncoPrint plots. ns. *p* ≥ 0.05; ^*^p < 0.05; ^**^p < 0.01; ^***^p < 0.001.

In this study, we investigated the expression of SART3 mRNA in various human cancers and corresponding normal tissues. Our analysis revealed that SART3 showed a significant increase in 13 types of cancer, including liver, esophageal, and pancreatic cancers (*p* < 0.001) (Figure 1A). To validate these findings in HCC, we used five independent datasets (TCGA, GSE14520, GSE76427, GSE121248 and GSE62232) to assess the transcriptome levels of SART3 in HCC tissues and paracancerous tissues. Our results demonstrated that the expression of SART3 was statistically higher in HCC tissues compared to paired/unpaired paraneoplastic tissues (*p* < 0.001) (Figure 1B). Moreover, SART3 was found to be a noteworthy biomarker for identifying HCC and paracancerous tissues in these five independent datasets (Supplementary Figure 1). We also confirmed these results using immunohistochemical staining of HCC and normal liver tissues (Figure 1C). Additionally, we investigated the genomic and copy number alterations of SART3 using the cBioPortal for Cancer Genomics website. Our analysis suggested that SART3 gene amplification, truncation mutations, and splicing mutations occurred at a frequency of 0.7% (Figure 1D).

### Elevated SART3 expression is an independent factor predicting poor prognosis in HCC patients

**Figure 2:**
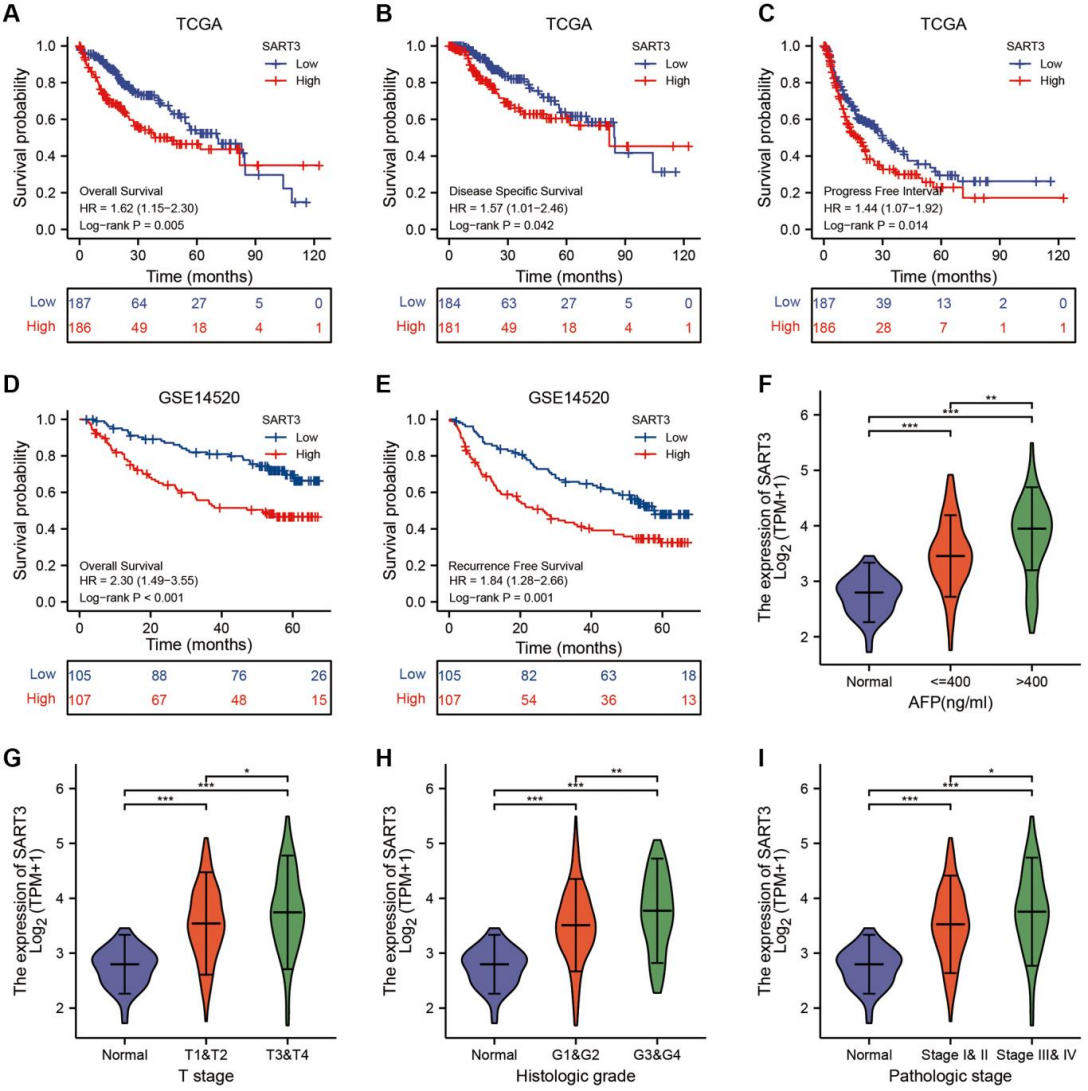
**High expression of SART3 correlates with poor prognosis and advanced staging of HCC.** Kaplan-Meier curves show SART3^high^ and SART3^low^ groups for (**A**) OS, (**B**) DSS and (**C**) PFS in TCGA dataset, and for (**D**) OS and (**E**) RFS in GSE14520 dataset. Violin plot shows SART3 expression at different AFP levels (**F**), T-stage (**G**), histological grade (**H**) and pathological stage (**I**) expression levels, ^*^p < 0.05; ^**^p < 0.01; ^***^p < 0.001.

**Figure 3:**
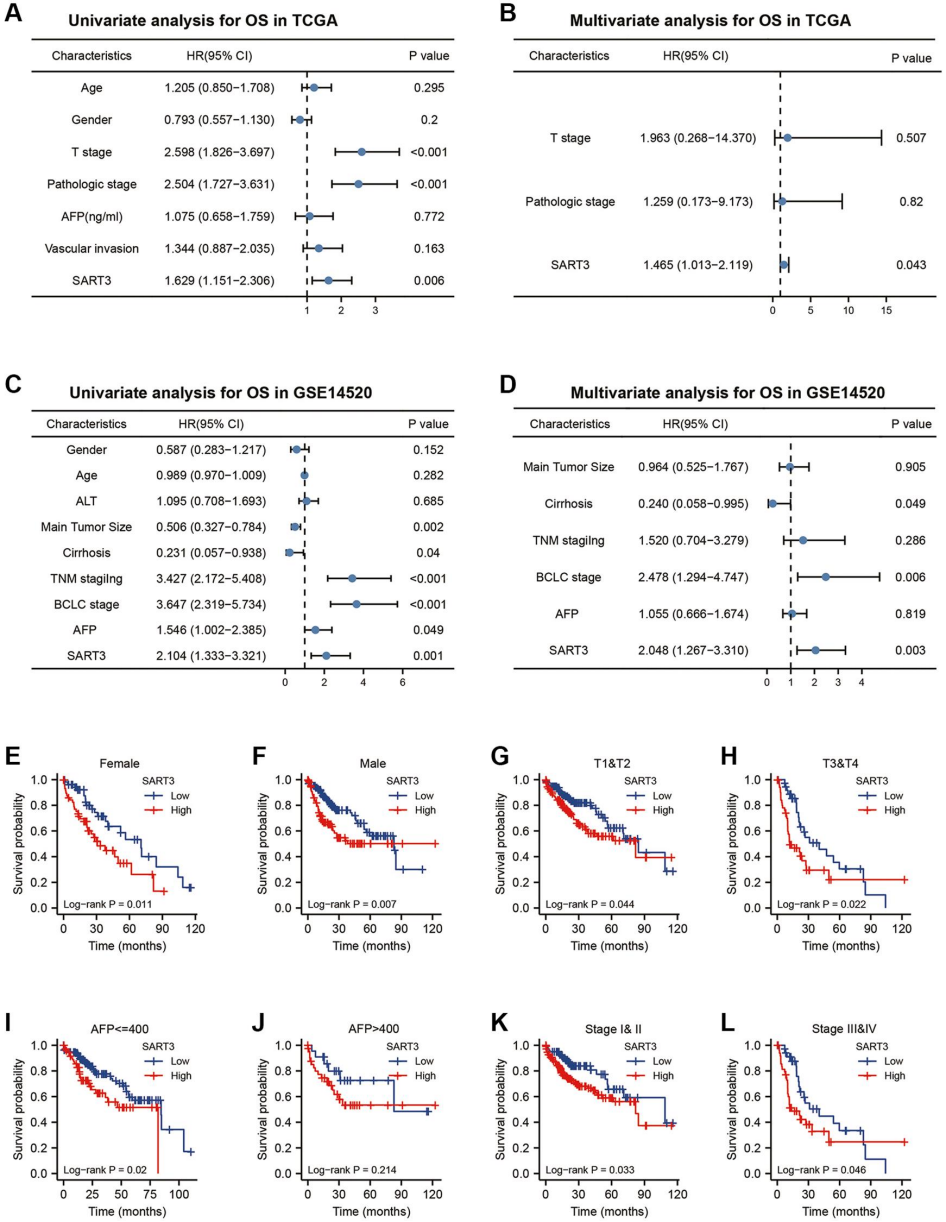
**Prognostic value of SART3 in HCC.** (**A**) Univariate Cox regression model and (**B**) multivariate Cox regression model in TCGA. (**C**) Univariate Cox regression model and (**D**) Multivariate Cox regression model in GSE14520. Kaplan-Meier survival curves for subgroups of SART3^high^ and SART3^low^ patients in (**E**) female, (**F**) male, (**G**) T1 and T2, (**H**) T3 and T4, (**I**) AFP ≤400, (**J**) AFP >400, (**K**) stage I and II and (**L**) stage III and IV.

To evaluate the impact of SART3 on patient survival in hepatocellular carcinoma (HCC), we conducted a comprehensive analysis of various datasets. In the TCGA dataset, we observed that SART3 overexpression (grouped at median) was significantly associated with poorer overall survival (OS) (*p* = 0.005), disease-specific survival (DSS) (*p* = 0.042) and progression-free interval (PFI) (*p* = 0.014) (Figure 2A–2C). Similarly, in the GSE14520 dataset, SART3 overexpression (grouped at median) was significantly associated with poorer OS (*p* < 0.001) and relapse-free survival (RFS) (*p* = 0.001) (Figure 2D, 2E). Furthermore, we analyzed the expression levels of SART3 in different stages of HCC and found that its overexpression was associated with advanced stages of HCC (Figure 2G–2I) and higher levels of AFP (Figure 2F). Univariate analysis in TCGA showed that SART3, T-stage, and pathological stage demonstrated a significant correlation with an unfavorable prognosis (Figure 3A). Similarly, in GSE14520, SART3 (low vs. high, HR 2.104, *p* = 0.001), main tumor size (>5 cm vs. ≤5 cm, HR 0.506, *p* = 0.002), cirrhosis (Yes vs. No, HR 0.231, *p* = 0.04), AFP (≥300 ng/ml vs. >300, HR 1.546, *P* = 0.049), TNM stage (I and II vs. III, HR 3.427, *p* < 0.001) and BCLC stage (stage 0 and A vs. B and C, HR 3.647, *p* < 0.001) were also significantly associated with poor prognosis (Figure 3C). Multivariate analysis in TCGA and GSE14520 showed that the high expression of SART3 was a standalone risk factor for HCC (*p* < 0.05) (Figure 3B, 3D), particularly in subgroups of male and female patients, different T stages, pathological stages, and AFP levels (Figure 3E–3L). In summary, our findings suggest that SART3 overexpression is an independent poor prognostic factor for HCC.

### Validation of SART3 using Guangxi patient samples

**Figure 4:**
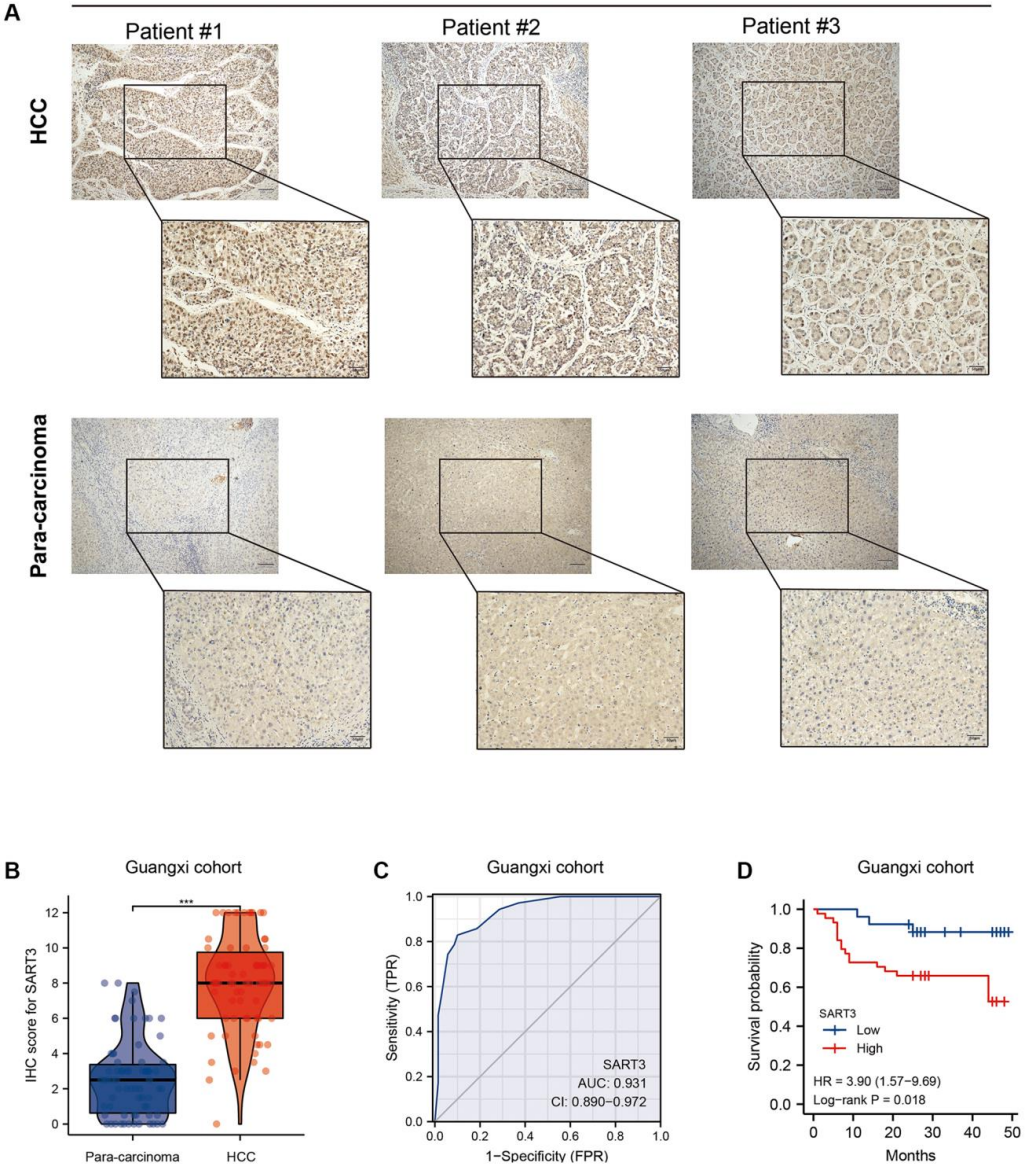
**Validation of SART3 in patients with HCC in Guangxi.** (**A**) Representative IHC images showing the *in situ* expression of SART3 in HCC and adjacent liver tissues. (**B**) Paired plots showing immunostaining scores of SART3 in HCC and adjacent liver tissues in the Guangxi cohort. (**C**) Diagnostic ROC curves of SART3 in the Guangxi cohort. (**D**) OS of SART3^high^ and SART3^low^ groups in the Guangxi cohort based on immunohistochemical staining scores.

In order to further validate the findings presented above, we conducted an immunohistochemical analysis on paired HCC and paraneoplastic tissues obtained from the First Affiliated Hospital of Guangxi Medical University (*n* = 70; baseline information is provided in Table 1). Our results demonstrate a significant increase in the nuclear expression of SART3 in HCC tissues compared to adjacent tissues (Figure 4A). This observation was confirmed by higher immunohistochemistry (IHC) scores in HCC tissues compared to adjacent tissues (*p* < 0.001) (Figure 4B). Remarkably, our results indicate that SART3 exhibits excellent diagnostic performance in the Guangxi cohort (AUC = 0.931) (Figure 4C), and that its overexpression is associated with a poorer prognosis for HCC patients (*p* = 0.018) (Figure 4D).

**Table 1:** Correlation between SART3 expression and clinicopathologic in Guangxi cohort.

**Variables**		**SART3 immunostaining score**		***X* ^2^**	***P* value**
		**Low (*n* = 26)**	**High (*n* = 44)**		
Gender	Male	23 (32.9%)	40 (57.1%)	0.000	1.000
	Female	3 (4.3%)	4 (5.7%)		
Age	>45	13 (18.6%)	19 (27.1%)	0.306	0.580
	≤45	13 (18.6%)	25 (35.7%)		
BMI	0 ≤ 28	24 (34.8%)	41 (59.4%)	0.000	1.000
	>28	2 (2.9%)	2 (2.9%)		
Cirrhosis	No	7 (10%)	7 (10%)	1.239	0.266
	Yes	19 (27.1%)	37 (52.9%)		
Child pugh	A	26 (37.1%)	42 (60%)	0.130	0.718
	B	0 (0%)	2 (2.9%)		
AFP	0 ≤ 400	18 (25.7%)	23 (32.9%)	1.937	0.164
	>400	8 (11.4%)	21 (30%)		
Tumorsize	≤5 cm	18 (26.1%)	20 (29%)	3.380	0.066
	>5 cm	8 (11.6%)	23 (33.3%)		
CNLC stage	Ia	14 (20.6%)	19 (27.9%)	7.683	0.053
	Ib	6 (8.8%)	12 (17.6%)		
	IIa	4 (5.9%)	3 (4.4%)		
	IIIa	0 (0%)	10 (14.7%)		
BCLC stage	A	21 (30.4%)	27 (39.1%)	3.082	0.214
	B	3 (4.3%)	6 (8.7%)		
	C	2 (2.9%)	10 (14.5%)		

Abbreviations: BMI: Body Mass Index; AFP: alpha fetoprotein; BCLC: Barcelona Clinic Liver Cancer; CNLC: China liver cancer staging.

### SART3 is associated with genomic alterations in HCC

**Figure 5:**
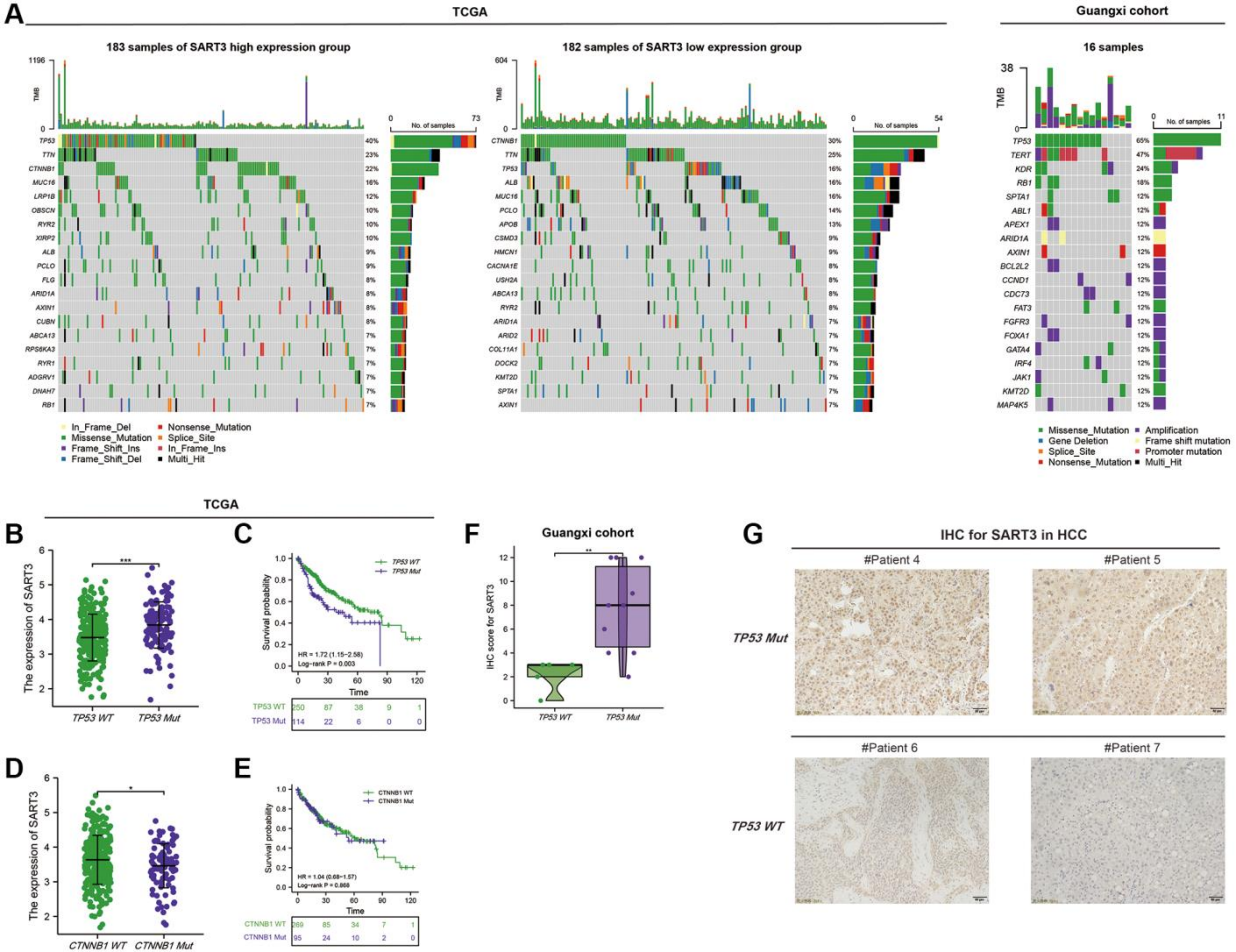
**Somatic mutation landscape of HCC based on SART3.** (**A**) Waterfall plots demonstrate the HCC somatic mutation landscape in the SART3^high^ and SART3^low^ in TCGA and Guangxi cohort. In the TCGA cohort, (**B**) SART3 expression between TP53 mutation and TP53 wild group, (**C**) survival analysis of TP53 mutation group and TP53 wild group, (**D**) SART3 expression between CTNNB1 mutation and wild group, (**E**) survival analysis of CTNNB1 mutation group and CTNNB1 wild group. (**F**) Immunohistochemical staining intensity of SART3 between TP53 mutation and TP53 wild group in the Guangxi cohort. (**G**) Representative images of immunohistochemistry.

To investigate the somatic mutation landscape of SART3 in HCC patients, we obtained somatic mutation data from HCC patients using the GDC data platform. We then utilized the maftools R package to visualize the results in mutation annotation format (MAF). Our analysis revealed the top 20 genes with the highest mutation frequency, including TP53 (40%), TTN (23%), CTNNB1 (22%), MUC16 (16%), LRP1B (12%), OBSCN (10%), RYR2 (10%), and XIRP2 (10%) in the SART3^high^ group, and CTNNB1 (30%), TTN (25%), TP53 (16%), ALB (16%), MUC16 (16%), PCLO (14%), APOB (13%) in the SART3^low^ group (Figure 5A). Notably, the mutation frequency of TP53 was significantly higher in the SART3^high^ group than in the SART3^low^ group (40% vs. 16%), whereas the mutation frequency of CTNNB1 was lower in the SART3^high^ expression group than in the SART3^low^ group (22% vs. 30%). To further elucidate the relationship between SART3 and TP53/CTNNB1 mutation, we examined the expression level of SART3 in the TP53/CTNNB1 mutation group and wild group, and performed survival analyses. Our results showed that the expression level of SART3 was significantly higher in the TP53 mutation group than in the wild group (*p* < 0.001) (Figure 5B), while the expression level was lower in the CTNNB1 mutation group than in the wild group (*p* = 0.037) (Figure 5D). Additionally, survival analysis revealed that the TP53 mutation group had worse survival (*p* = 0.003) (Figure 5C), while the CTNNB1 mutation was not associated with patient survival time (*p* = 0.868) (Figure 5E). Furthermore, we verified our results by immunohistochemistry in the Guangxi cohort, which revealed that the expression of SART3 was significantly elevated (*p* = 0.009) in the TP53 mutation group (Figure 5F), as demonstrated in representative images in Figure 5G.

### SART3 can upregulate the expression of immune checkpoints

**Figure 6:**
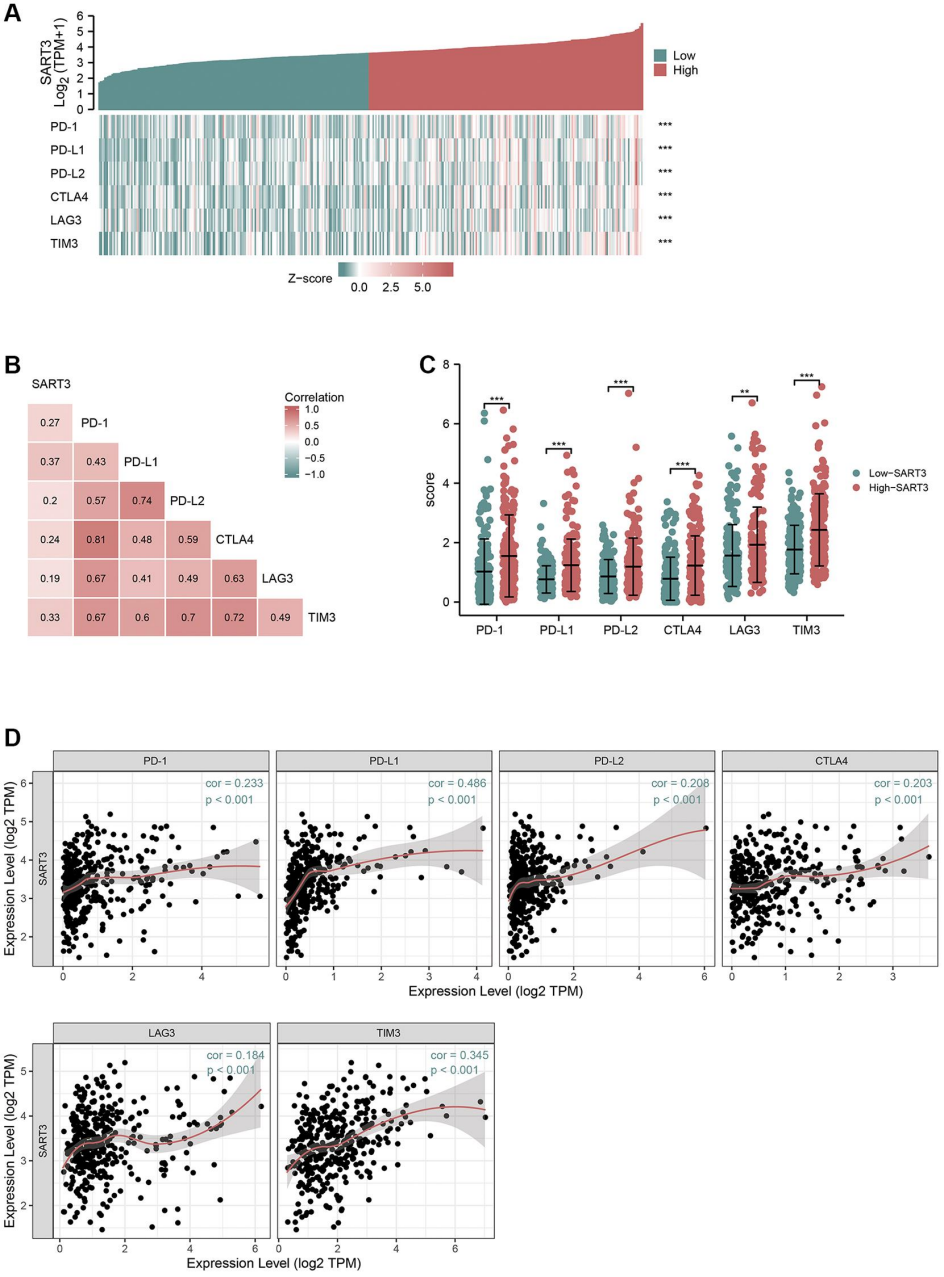
**High expression of SART3 upregulates the expression level of immune checkpoint genes.** (**A**) Heat map showing co-expression of SART3 and immune checkpoint genes. (**B**) Correlation matrix showing SART3-immune checkpoint association. (**C**) Expression levels of immune checkpoint genes between SART3 high and SART3 low groups. (**D**) Correlation of SART3 with immune checkpoint genes was verified in TIMER database.

Several studies have demonstrated that mutations in the TP53 gene can upregulate the expression of immune checkpoints [29]. Immune checkpoint inhibitors (ICIs) have shown tremendous benefits in cancer patients by prolonging their survival; however, the efficacy of ICIs is contingent upon the expression of immune checkpoint genes [30]. As a result, we investigated the correlation between SART3 and immune checkpoint genes in HCC. Co-expression heat maps and correlation matrices showed that SART3 had a significant correlation with PD-L1 (r > 0.3), TIM-3 (r > 0.3), PD-1, PD-L2, CTLA4, and LAG3 (Figure 6A, 6B). Furthermore, we observed upregulation of these genes in the SART3^high^ group (*p* < 0.001) (Figure 6C). Additionally, we performed further correlation analysis through the TIMER website and found that SART3 has a significantly positive association with PD-L1 and TIM3 (Figure 6D), which is consistent with the TCGA results.

### The role of SART3 in immune infiltration of HCC

**Figure 7:**
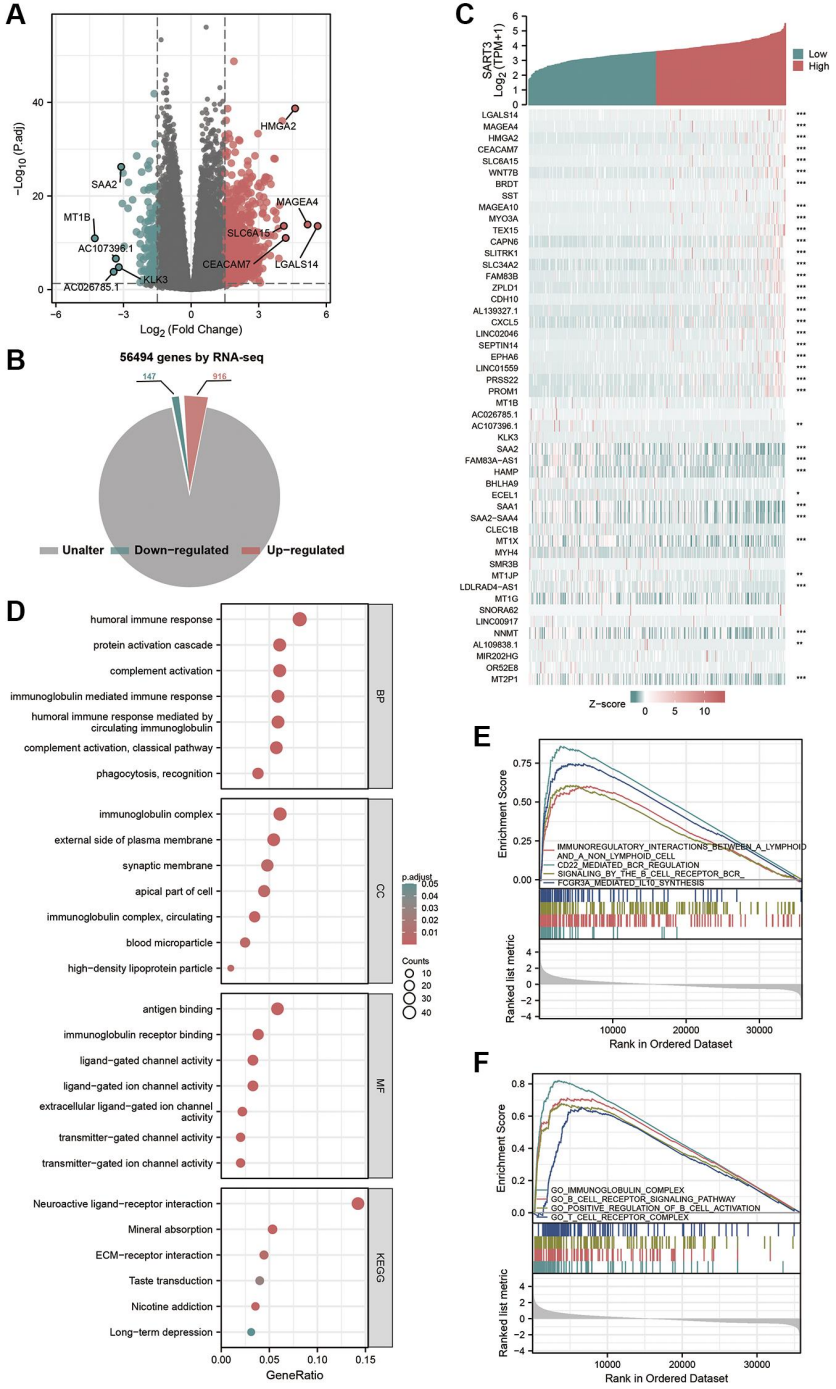
**Identification and functional annotation of DEGs and GSEA analysis.** (**A**) Volcano plot showing DEGs between SART3 high and SART3 low groups. (**B**) Pie chart showing the percentage of up-regulated DEGs and down-regulated DEGs in transcriptome genes. (**C**) Heat map showing the top 25 up- and down-regulated genes. (**D**) Bubble plots of DEGs significantly enriched in GO terms and KEGG pathways. Enrichment plots showing (**E**) four significantly positively correlated KEGG pathways and (**F**) four significantly positively correlated GO annotations.

To investigate the potential mechanisms underlying the role of SART3 in HCC, we identified 1063 DEGs (|log2FoldChange| >1.5, *p* < 0.05) between samples with high and low SART3 expression in the TCGA LIHC cohort (Figure 7A). Of these, 916 genes were up-regulated and 147 genes were down-regulated (Figure 7B), and we demonstrated the top 25 up/down-regulated genes using a heat map (Figure 7C). Functional enrichment analysis of GO and KEGG of the DEGs revealed that SART3 is involved in immune infiltration-related pathways of HCC, such as the classical pathway of complement activation, humoral immune response mediated by circulating immunoglobulin, immunoglobulin receptor binding, antigen binding, and immunoglobulin-mediated immune response (Figure 7D). Additionally, the results of GSEA analysis indicated that SART3 was closely associated with immune infiltration, such as immunomodulatory interactions between lymphocytes and non-lymphoid cells, signaling of the B-cell receptor RCR, immunoglobulin complex, B-cell receptor signaling pathway, and T-cell receptor complex (Figure 7E, 7F). The results of the DEGs enrichment analysis and GSEA suggest that SART3 may influence HCC invasion and metastasis through immune cell infiltration and response in tumors.

**Figure 8:**
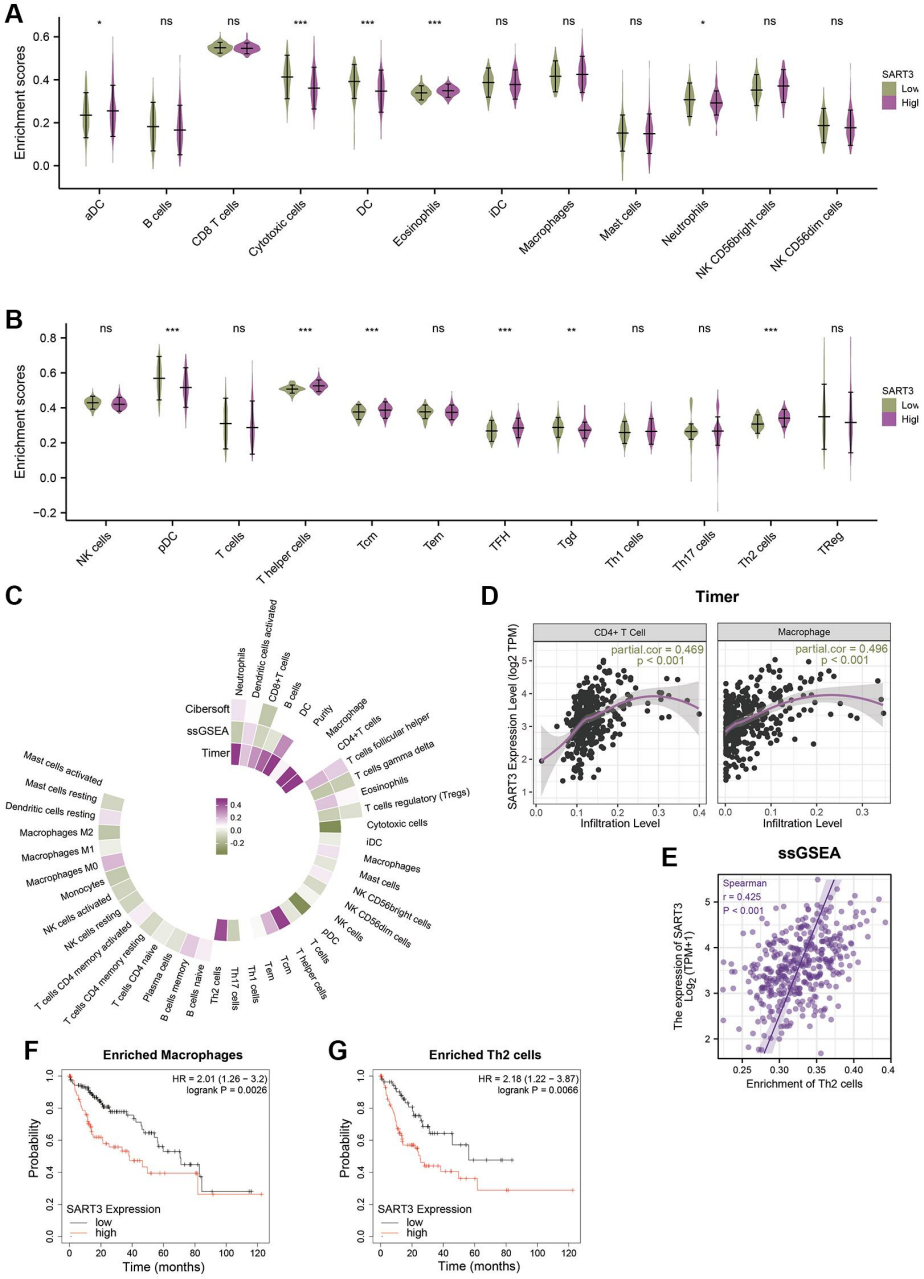
**Correlation between SART3 expression and immune infiltration in HCC and its impact on prognosis.** (**A**, **B**) Violin plots showing the degree of infiltration of different immune cells in the SART3^high^ and SART3^low^ groups, ns: *p* ≥ 0.05; ^*^p < 0.05; ^**^p < 0.01; ^***^p < 0.001. (**C**) Circle heat map showing the correlation between SART3 and different immune cells. (**D**, **E**) Scatter plot showing the correlation between SART3 expression levels and CD4+ T cells, macrophages and Th2 cells. Subgroup survival analysis of the SART3^high^ and SART3^low^ groups in (**F**) enriched macrophages and (**G**) enriched Th2 cells.

To further elucidate the role of SART3 in HCC immune infiltration, we employed ssGSEA, Cibersort, and TIMER to identify the relationship between SART3 and immune infiltration. Our analysis revealed that the high SART3 group had higher infiltration of Th2 cells, activated DCs, follicular helper T cells, eosinophils, central memory T cells, and T helper cells, while the low SART3 group had more infiltrated cytotoxic cells, DCs, neutrophils, pDCs, and Tgd (Figure 8A, 8B). We further demonstrated the correlation of SART3 with multiple immune cells using a circular heat map and found that SART3 was closely associated with multiple immune cells (Figure 8C), such as CD4+ T cells, macrophages, and Th2 cells (*p* < 0.05) (Figure 8D, 8E). To investigate whether SART3 affects the survival of HCC patients through immune infiltration, we performed survival analysis of relevant immune cell subsets using the K-M plotter website. The results showed that the prognosis was worse in the SART3 high expression group in HCC with increased infiltration of Th2 cells and macrophages (*p* < 0.05) (Figure 8F, 8G). These results suggest that SART3 may influence the survival of HCC patients through immune infiltration.

### SART3 and its related genes regulate RNA transcription in HCC

**Figure 9:**
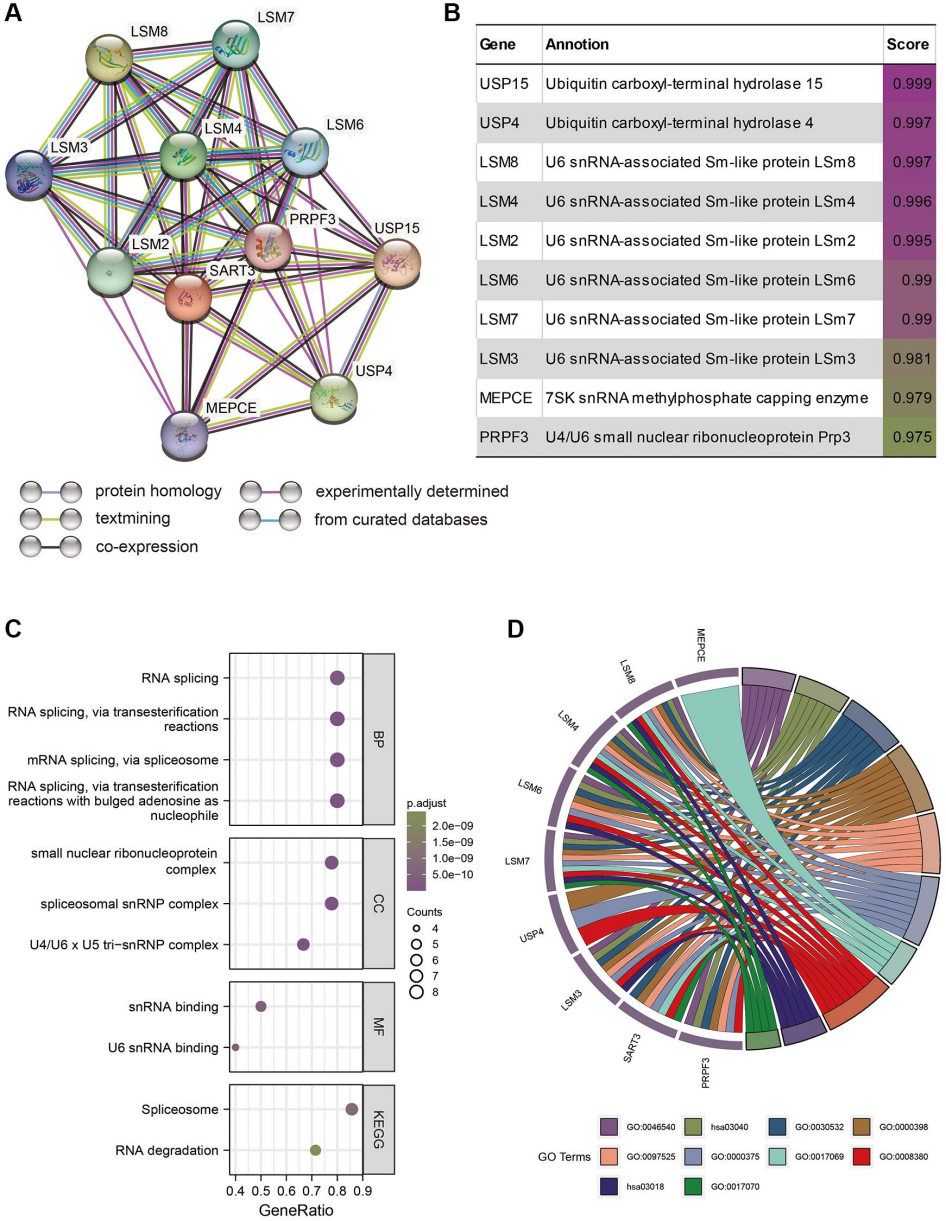
**SART3 and its related genes are closely associated with RNA transcription in HCC cells.** (**A**) PPI network of SART3-associated genes. (**B**) Annotations and correlation coefficients of nine SART3-related genes. (**C**, **D**) Enriched GO and KEGG pathways of SART3 and its related genes.

We investigated the role of SART3 and its associated genes in hepatocellular carcinoma (HCC) by identifying nine genes that had the strongest correlation with SART3 using the STRING website. These genes are USP15, USP4, LSM8, LSM4, LSM2, LSM6, LSM7, LSM3, and MEPCE (Figure 9A, 9B). Our GO and KEGG analyses revealed that SART3 is significantly associated with the regulation of RNA transcription, particularly RNA splicing, spliceosomal snRNP complex, U4/U6 × U5 tri-snRNP complex, and Spliceosome (Figure 9C, 9D). To investigate whether SART3 affects the prognosis of HCC patients by regulating RNA transcription, we conducted expression tests and survival analyses of these genes in TCGA. According to our findings, HCC tissues exhibited significant upregulation of all nine genes compared to paraneoplastic tissues (*p* < 0.001) (Supplementary Figure 2A). Additionally, the upregulation of the expression of eight genes, except for USP4, was associated with worse OS in patients (Supplementary Figure 2B–2I). In conclusion, our findings suggest that SART3 may play a significant role in the survival of HCC patients by regulating RNA transcription.

### Effect of SART3 on the proliferation and migration of HCC cells

**Figure 10:**
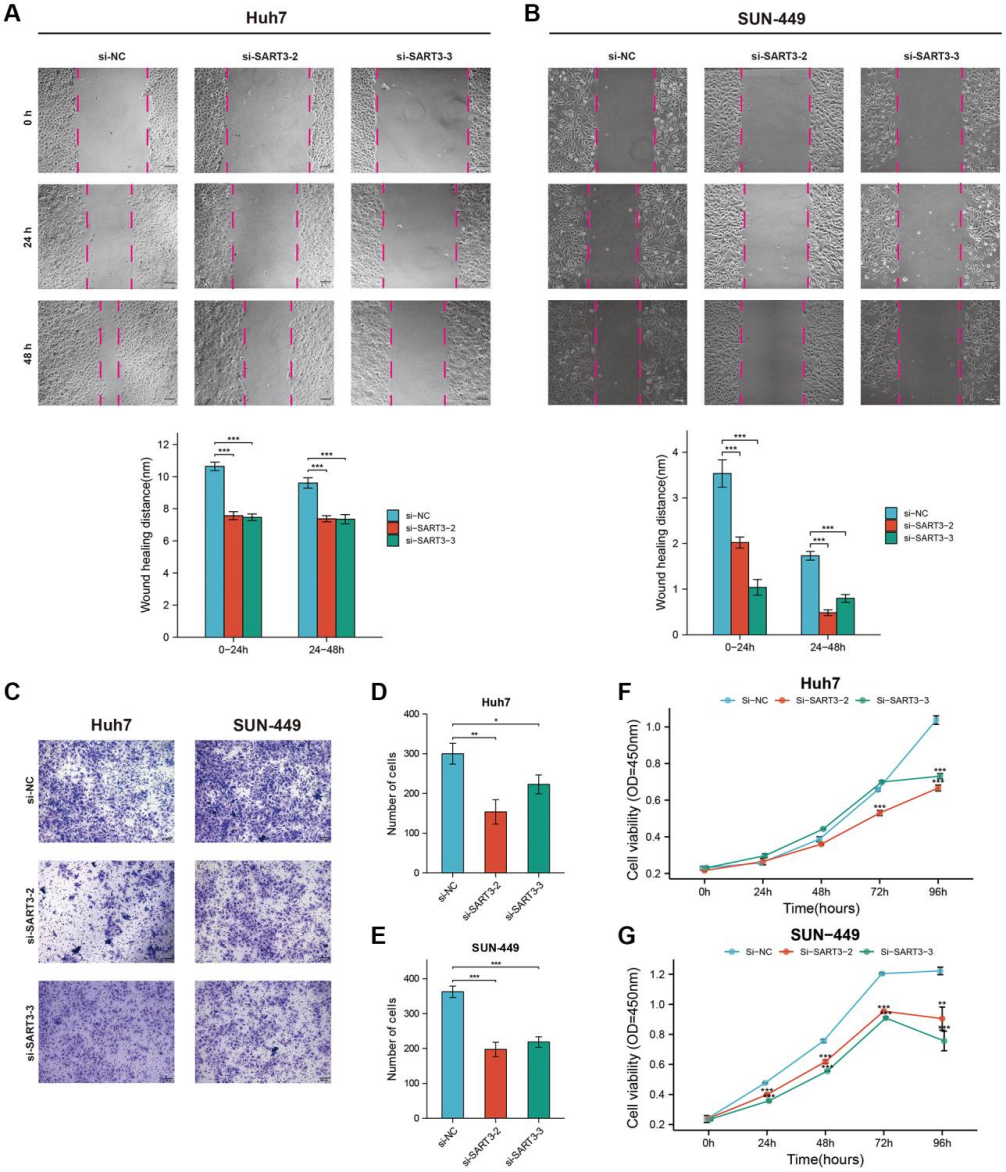
**Representative images and corresponding histograms of the wound healing assay of Huh-7.** (**A**) and SUN-449 (**B**) cells in si-SART3 group and control group. Representative images (**C**) and corresponding histograms (**D**, **E**) of the Trans-well assay of Huh-7 and SUN-449 cells in si-SART3 group and control group. Growth curves of Huh-7 (**F**) and SUN-449 (**G**) cells in si-SART3 group and control group by CCK-8 assay.

We quantified SART3 expression levels in various liver cancer cell lines through qPCR analysis and observed that Huh-7 and SUN-449 had the highest SART3 expression (Supplementary Figure 3A). Consequently, we selected Huh-7 and SUN-449 cells to study the effects of SART3 downregulation with siRNA technology, which was verified through qPCR analysis (Supplementary Figure 3B, 3C). Our results from the Wound-Healing and Trans-well assays indicated that inhibition of SART3 expression reduced the migratory ability of HCC cells (Figure 10A–10E). Moreover, the CCK8 assay revealed that SART3 downregulation impeded the proliferation ability of HCC cells (Figure 10F, 10G).

### Identifying potential therapeutic agents for HCC based on SART3 expression

**Figure 11:**
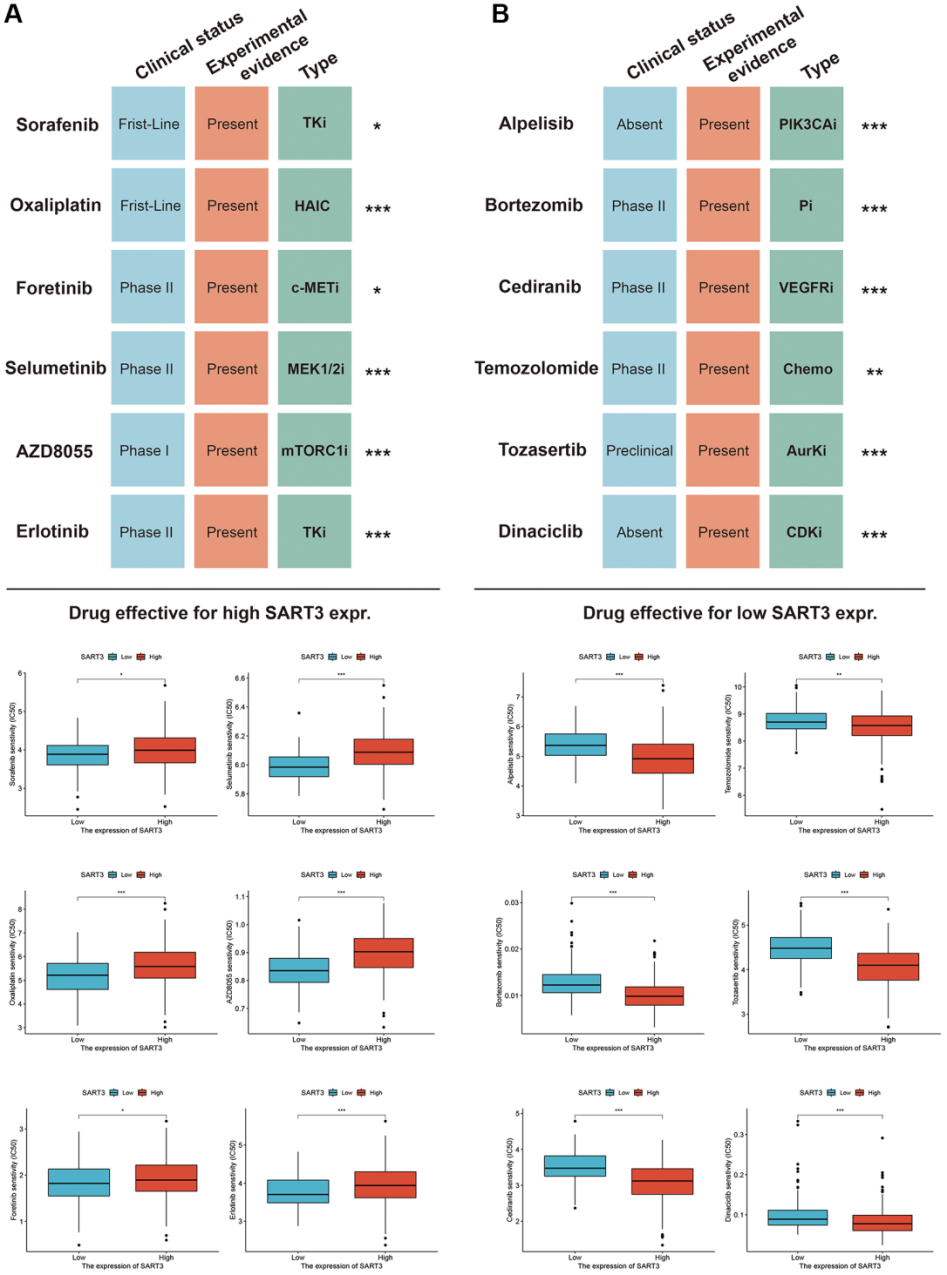
**Sensitivity drugs for SART3 high and low expression groups in HCC.** Sensitivity drugs for SART3 high expression (**A**) and SART3 low expression (**B**). Abbreviations: TKi: Tyrosine kinase inhibitor; HAIC: hepatic arterial infusion chemotherapy; VEGFRi: vascular endothelial growth factor receptor inhibitor; c-METi: c-MET inhibitor; MEK1/2i: mitogen-activated protein kinase 1 and 2 inhibitor; Chemo: Chemotherapy; AurKi: pan-aurora kinase inhibitor; CDKi: Cyclin-dependent kinase inhibitor; mTORC1i: mTOR Complex 1 inhibitor; Pi: proteasome inhibitor.

In this study, we aimed to explore the clinical therapeutic value of SART3 for HCC by identifying potentially sensitive therapeutic agents through the GDSC database. We used high and low SART3 expression as criteria for selecting the drugs. The results indicated that the following drugs were sensitive for the high SART3 expression group: Sorafenib, Oxaliplatin, Foretinib, Selumetinb, AZD8055, and Erlotinib (Figure 11A). For the low SART3 expression group the sensitive drugs were alpelisib, bortezomib, cediranib, temozolomide, tozasertib, and dinaciclib (Figure 11B). To gain further insight into the therapeutic value of these drugs in HCC, we searched PubMed (https://www.ncbi.nlm.nih.gov/pubmed/) and the Clinical Trials Registry (https://www.clinicaltrials.gov/) to compile a list of the drugs and investigate their therapeutic targets, experimental evidence, and clinical trial stages. Our findings are presented in Figure 11.

## DISCUSSION

The hepatocellular carcinoma (HCC) is a highly heterogeneous tumor [31]. Drug resistance, susceptibility to metastasis, and high recurrence rate contribute to poor prognosis for HCC patients. Molecular subtype classification has been used in several studies to address HCC heterogeneity. Calderaro et al. [32] research classified HCC into two major categories: proliferative and non-proliferative, with TP53 mutations belonging to the former and CTNNB1 mutations to the latter. Our study investigates the differential expression of SART3 in the mutant and non-mutant groups of TP53 and CTNBB1, providing insights into the molecular typing of HCC.

TP53 is the most frequently mutated gene in human cancers [33], and p53’s oncogenic role is critical in the regulation of cell cycle, cell death, and transcription factors. Mutation of TP53 impairs the function of p53, leading to cancer development by regulating other transcription factors [34]. In our study, we found that SART3 expression was significantly upregulated in TP53-mutated HCC and correlated with poor prognosis of patients. Our findings suggest that SART3 may be a downstream gene regulated by p53, and its regulatory function is impaired after TP53 mutation leading to upregulation of SART3. TP53 mutations are predictive of immunotherapy efficacy [35] and upregulate immune checkpoint expression. Immunosuppressive agent’s efficacy correlates with the expression of immune checkpoints [29]. We further investigated the correlation between SART3 and immune checkpoints, and our results indicate that high expression of SART3 significantly upregulates the expression of immune checkpoints.

Tumorigenesis, invasion, and metastasis are tightly linked to the tumor microenvironment (TME), which comprises stromal cells, such as fibroblasts, and infiltrating immune cells, such as macrophages [36, 37]. The immune response to tumor cells can have a dual effect, activating both anti-tumor pathways and creating an immunosuppressive microenvironment [38]. Research has demonstrated that macrophage infiltration in tumor tissue leads to poor patient prognosis and is associated with resistance to chemotherapeutic agents in most cancers [39]. The main function of Th2 cells is to suppress T cell activation, allowing the body to accept foreign cells. The TH1/TH2 balance is crucial, and when Th2 cells over suppress Th1 cells, the body becomes susceptible to tumor development [40]. Additionally, some studies have revealed that oncogenes can regulate immune infiltration in tumor cells [41]. In our study, by conducting enrichment analysis of GSEA and identifying DEGs, we found that SART3 was significantly enriched in the immune infiltrative pathway. Moreover, we discovered that it is closely associated with several immune cells, such as Th2 cells and macrophages, and may affect patient prognosis through them.

Tumor cells, unlike normal cells, are characterized by excessive proliferation, abnormal differentiation, and impaired apoptosis. Gene transcription and cell cycle play crucial roles in these processes. Our study showed that SART3 is significantly associated with gene transcription regulation and cell cycle, and may contribute to the poor prognosis of HCC patients through these mechanisms. Furthermore, our cell function experiments indicated that SART3 could promote the proliferation and migration of HCC cells.

In 2007, the introduction of sorafenib, a targeted drug, instilled new hope for HCC patients [42]. Over recent years, with the advancements in research and drug development, various therapeutic agents and modalities have been utilized for the treatment of advanced HCC, including targeted therapy, immunotherapy, and hepatic artery infusion chemotherapy (HAIC). The combination of these therapeutic modalities has emerged as the primary treatment for advanced HCC, significantly prolonging the survival of patients [43]. Our study reveals that high and low SART3 expression levels can be used to select potentially responsive HCC therapeutic agents, including sorafenib, bortezomib, and oxaliplatin [44] (a first-line HAIC therapeutic agent for advanced HCC). Additionally, this study revealed that SART3 upregulates the expression of immune checkpoints, which may enhance the effectiveness of immunotherapy. These findings provide a new understanding of the clinical management of HCC.

In summary, our study analyzed multiple databases and cell function experiments to investigate the role of SART3 in HCC and how it affects patient prognosis. The findings indicate that SART3 may be a downstream gene of p53 and could serve as a novel biomarker for the diagnosis and prognosis of HCC. Moreover, SART3 was significantly associated with tumor immune infiltration in HCC and could upregulate the expression of immune checkpoints, making it a potential molecular target for future immunotherapy and targeted therapy in HCC. However, there are some limitations in our study. Firstly, the mechanism of SART3 regulation of immune infiltration needs to be clarified through animal experiments. Secondly, how P53 regulates SART3 requires further in-depth mechanistic studies.

## Supplementary Materials

Supplementary Figures

Supplementary Table 1
