# Supplementary material for: SART3, regulated by p53, is a biomarker for diagnosis, prognosis and immune infiltration in hepatocellular carcinoma
Source: Aging (Albany NY). 2023 Aug 24;15(16):8408–32. doi: 10.18632/aging.204978 (PMC10496991; doi:10.18632/aging.204978)
Supplement: Supplementary Figures [file aging-15-204978-s001.pdf]

SUPPLEMENTARY FIGURES

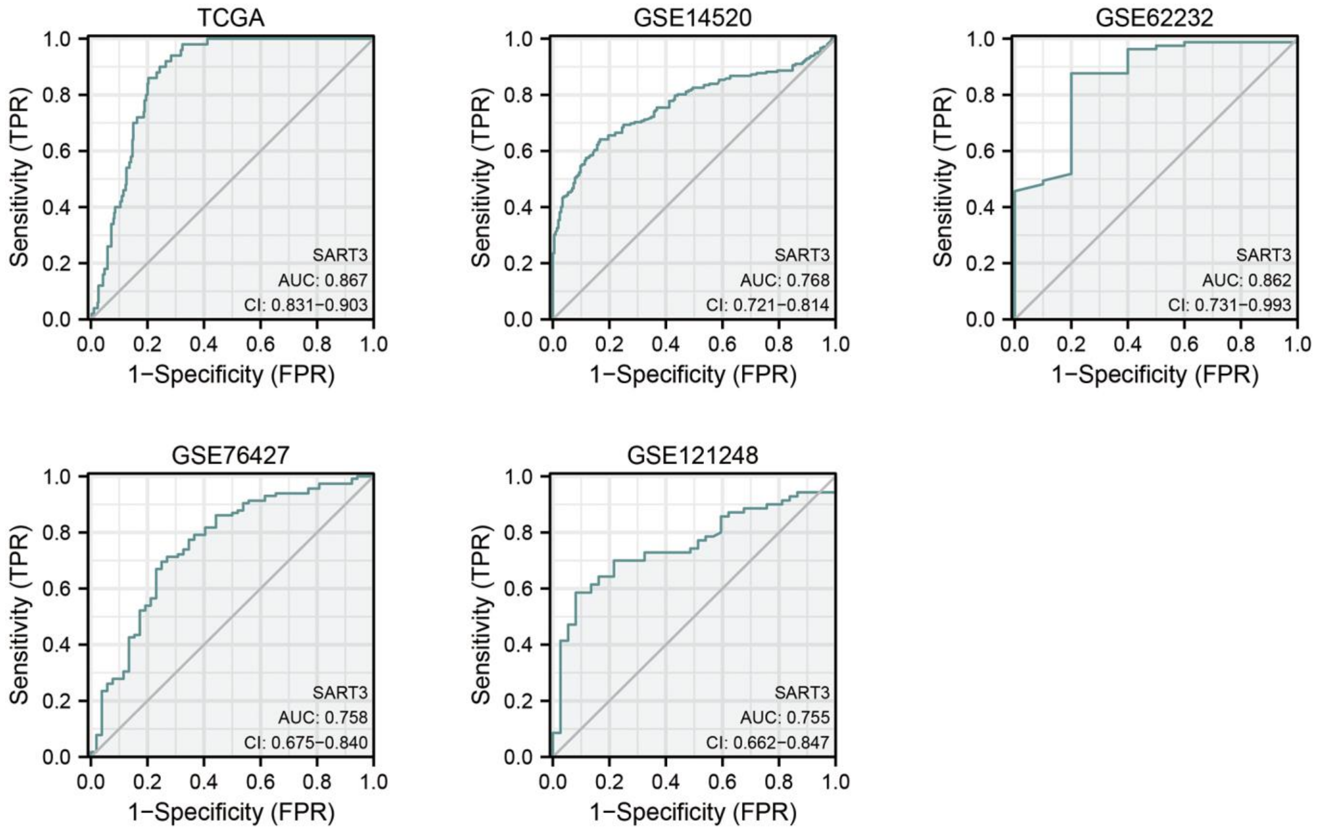

Supplementary Figure 1. Ability of SART3 to diagnose and predict survival in HCC. Diagnostic ROC curves of TCGA, GSE14520, GSE62232, GSE76427 and GSE121248.

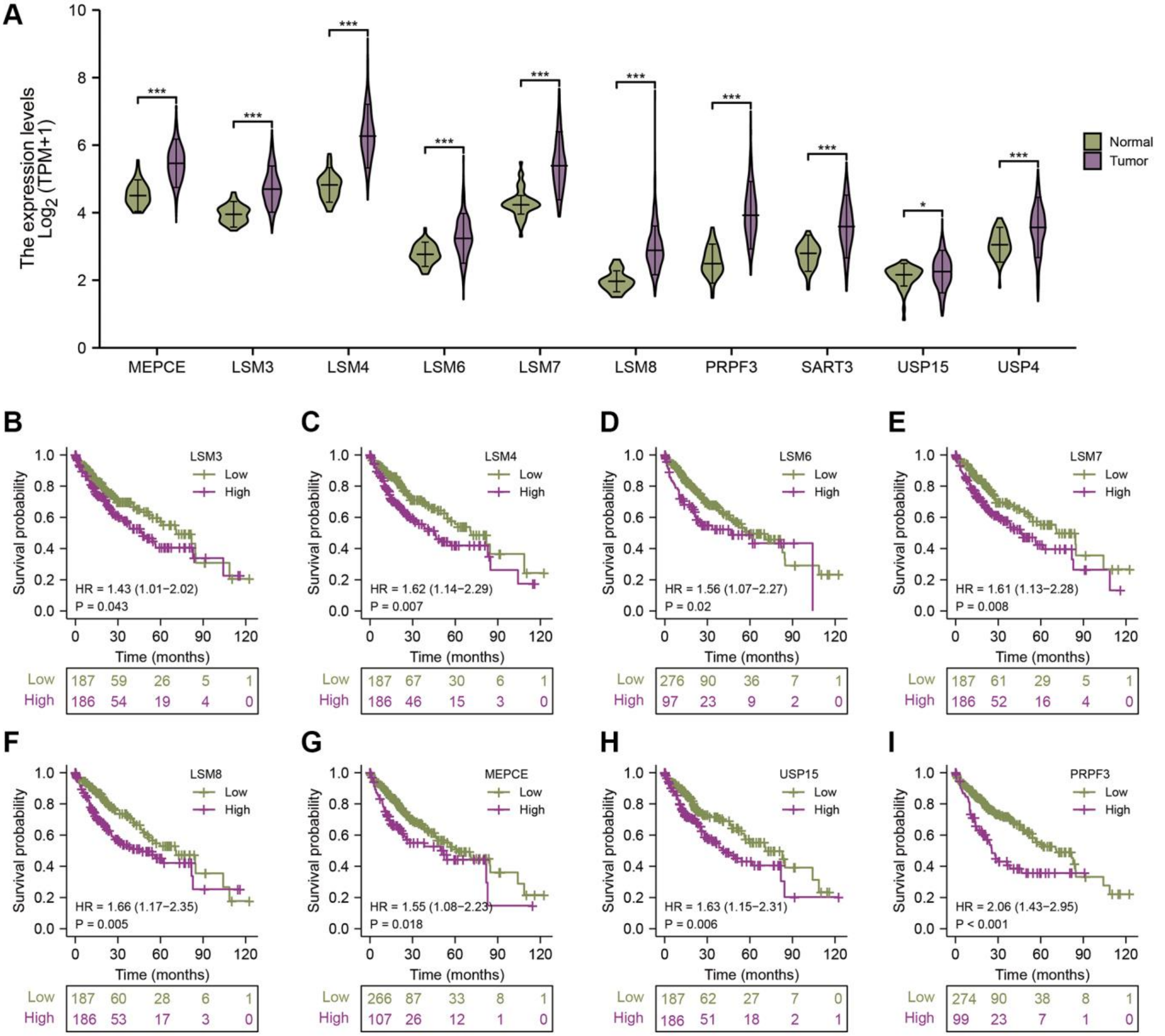

Supplementary Figure 2. Expression and prognostic significance of SART3-related genes in HCC. (**A**) Expression levels of the nine functional partners of SART3 in HCC and paraneoplastic tissues. survival curves of HCC patients in the TCGA cohort, classified according to the expression of (**B**) LSM3, (**C**) LSM4, (**D**) LSM6, (**E**) LSM7, (**F**) LSM8, (**G**) MEPCE, (**H**) USP15 and (**I**) PRPF3. $^{*}p < 0.05$; $^{***}p < 0.001$.

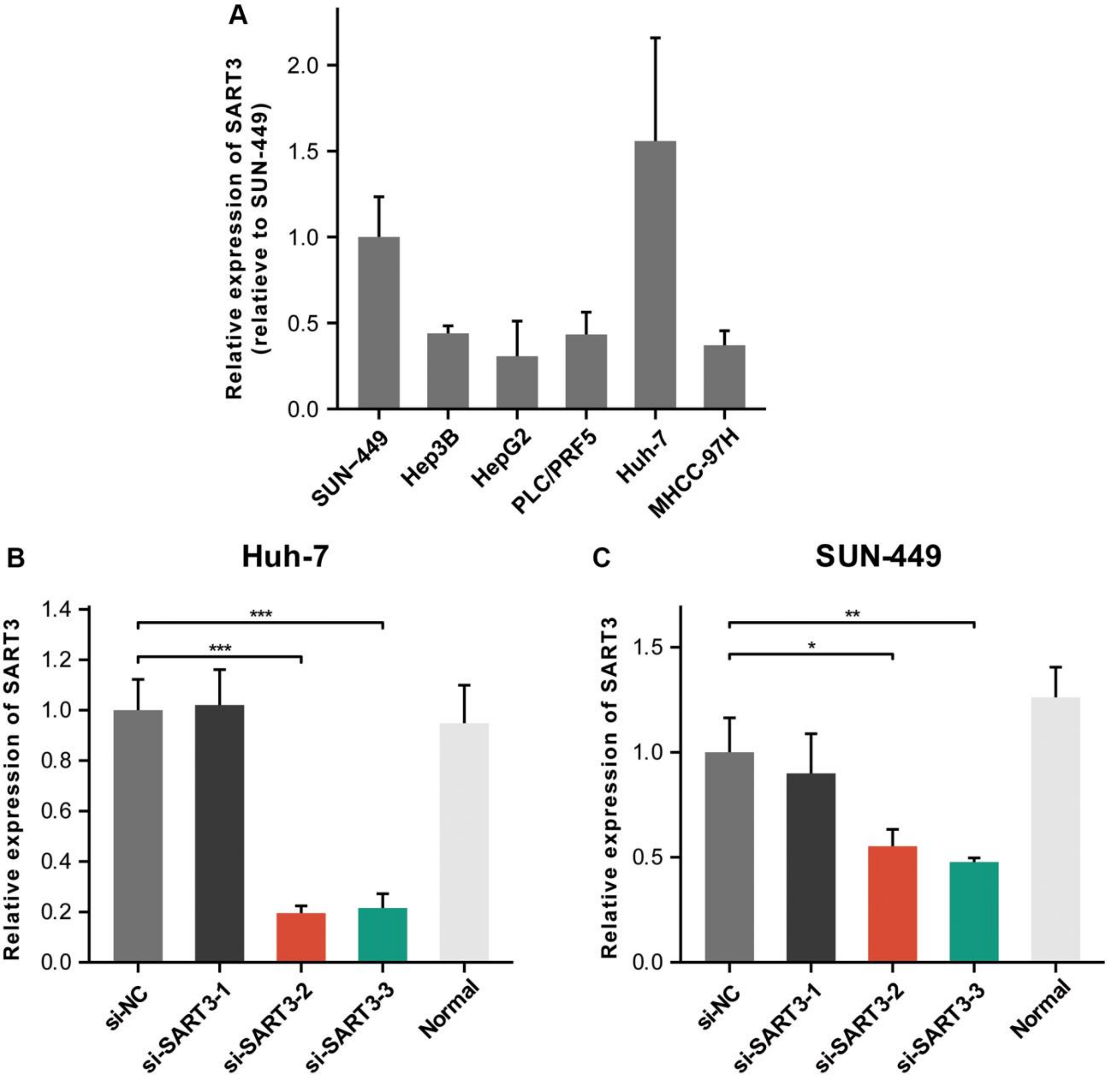

Supplementary Figure 3. Identification of down-regulation effects of SART3. (**A**) Expression of SART3 in several HCC cell lines. The relative expression of SART3 in Huh-7 (**B**) and SUN-449 (**C**) cells, respectively, in the si-SART3 group and control group, as determined by qPCR assay. $^{*}p < 0.05$; $^{**}p < 0.01$; $^{***}p < 0.001$.
