# Supplementary material for: SART3, regulated by p53, is a biomarker for diagnosis, prognosis and immune infiltration in hepatocellular carcinoma
Source: Aging (Albany NY). 2023 Aug 24;15(16):8408–32. doi: 10.18632/aging.204978 (PMC10496991; doi:10.18632/aging.204978)
Supplement: Supplementary Table 1 [file aging-15-204978-s002.pdf]

SUPPLEMENTARY TABLE

Supplementary Table 1. Sequence of interfering RNA for SART3.

Oligo name		Sequence (5′–3′)
SART3-Homo-si1	Sense strand	GCCAAGCUGUUUCUGAGAATT
	Antisense strand	UUCUCAGAAACAGCUUGGCTT
SART3-Homo-si2	Sense strand	GGACAGCAUCACCGUCUUUTT
	Antisense strand	AAAGACGGUGAUGCUGUCCTT
SART3-Homo-si3	Sense strand	GCCAGAAGAUGAGUGAAAUTT
	Antisense strand	AUUUCACUCAUCUUCUGGCTT
Negative control-FAM	Sense strand	UUCUCCGAACGUGUCACGUTT
	Antisense strand	ACGUGACACGUUCGGAGAATT-FAM
